# Rotational thromboelastometry results are associated with care level in COVID-19

**DOI:** 10.1007/s11239-020-02312-3

**Published:** 2020-10-17

**Authors:** Lou M. Almskog, Agneta Wikman, Jonas Svensson, Michael Wanecek, Matteo Bottai, Jan van der Linden, Anna Ågren

**Affiliations:** 1 Department of Anaesthesiology and Intensive Care, Capio St Göran’s Hospital, Stockholm, Sweden; 2 Department of Molecular Medicine and Surgery, Karolinska Institutet, Stockholm, Sweden; 3 Department of Clinical Immunology and Transfusion Medicine, Karolinska University Hospital and Department of CLINTEC, Karolinska Institutet, Stockholm, Sweden; 4 Department of Clinical Neuroscience, Karolinska Institutet, Stockholm, Sweden; 5 Department of Physiology and Pharmacology, Karolinska Institutet, Stockholm, Sweden; 6 Division of Biostatistics, Institute of Environmental Medicine, Karolinska Institutet, Stockholm, Sweden; 7 Perioperative Medicine and Intensive Care, Karolinska University Hospital, Stockholm, Sweden; 8 Coagulation Unit, Division of Hematology and Department of Molecular Medicine and Surgery, Karolinska Institutet, Karolinska University Hospital, 171 76 Stockholm, Sweden; 9 Department of Clinical Sciences, Danderyd Hospital, Stockholm, Sweden

**Keywords:** Covid-19, Thromboelastometry, Coagulopathy, Thrombosis, Fibrinogen

## Abstract

High prevalence of thrombotic events in severely ill COVID-19 patients have been reported. Pulmonary embolism as well as microembolization of vital organs may in these individuals be direct causes of death. The identification of patients at high risk of developing thrombosis may lead to targeted, more effective prophylactic treatment. The primary aim of this study was to test whether rotational thromboelastometry (ROTEM) at admission indicates hypercoagulopathy and predicts the disease severity, assessed as care level, in COVID-19 patients. The study was designed as a prospective, observational study where COVID-19 patients over 18 years admitted to hospital were eligible for inclusion. Patients were divided into two groups depending on care level: (1) regular wards or (2) wards with specialized ventilation support. Conventional coagulation tests, blood type and ROTEM were taken at admission. 60 patients were included; age 61 (median), 67% men, many with comorbidities (e.g. hypertension, diabetes). The ROTEM variables Maximum Clot Firmness (EXTEM-/FIBTEM-MCF) were higher in COVID-19 patients compared with in healthy controls (p < 0.001) and higher in severely ill patients compared with in patients at regular wards (p < 0.05). Our results suggest that hypercoagulopathy is present early in patients with mild to moderate disease, and more pronounced in severe COVID-19 pneumonia. Non-O blood types were not overrepresented in COVID-19 positive patients. ROTEM variables showed hypercoagulopathy at admission and this pattern was more pronounced in patients with increased disease severity. If this feature is to be used to predict the risk of thromboembolic complications further studies are warranted.

**Electronic supplementary material:**

The online version of this article (10.1007/s11239-020-02312-3) contains supplementary material, which is available to authorized users.

## Key points

- High prevalence of thrombosis is reported in Covid-19.
- Rotational thromboelastometry (ROTEM) can be a marker for hypercoagulopathy.
- In a single-center study of Covid-19 patients, ROTEM analysis showed signs of hypercoagulopathy.
- ROTEM hypercoagulopathy was more pronounced in severely ill patients.
- The risk of thrombosis and disease severity could possibly be predicted by early evaluation of ROTEM results.

## Background

Corona virus disease 2019 (COVID-19), caused by the severe acute respiratory syndrome coronavirus 2 (SARS-CoV-2), has taken hold of the world and spread globally with presently almost 35 million confirmed cases [1]. The lungs are the main target organ for COVID-19, and patients who become critically ill due to the virus generally suffer from respiratory distress leading to difficulties in ventilation and oxygenation. However, the respiratory symptoms in COVID-19 display atypical features, which have challenged the conventional mechanical ventilation strategies [2].

Studies of COVID-19 positive patients treated at Intensive Care Units (ICUs) show a cumulative incidence of thrombotic events of almost 50%, mainly pulmonary embolism [3, 4]. Autopsy reports have confirmed and even exceeded this high rate of thrombosis [5, 6], and a high prevalence of microthrombi in small veins of the lungs [7]. Thromboembolic complications may be a direct cause of death in COVID-19 [5, 6] and in addition, the loss of perfusion caused by thrombi in the lungs will impair pulmonary gas exchange independently of the direct tissue damage induced by the viral pneumonia. This will contribute to the respiratory failure that is the main cause of critical disease in patients with COVID-19. As a consequence, anticoagulant prophylaxis is recommended early in hospital care [8].

Elevated levels of fibrin degradation products (e.g. D-dimer) have consistently been reported as a strong prognostic factor associated with poor outcome in patients with COVID-19 [9, 10]. However, the D-dimer increase is not evident in early stages of the disease [10], limiting its usefulness as a prognostic marker. Maximum Clot Firmness (MCF) calculated using rotational thromboelastometry (ROTEM) is considered a good marker for hypercoagulopathy, which theoretically may be affected earlier during the disease course compared with D-dimer [11]. Studies assessing ROTEM in COVID-19 patients treated at ICUs have shown elevated MCF values [12, 13]. It is therefore possible that ROTEM variables may be of greater value as predictors of disease severity. However, no ROTEM data at earlier stages of the disease have, to our knowledge, yet been reported.

The primary aim of this study was to assess the presence of coagulopathy, measured by ROTEM, in COVID-19 patients at hospital admission, and evaluate whether a more pronounced coagulopathy may possibly reflect an increased need of ventilation support provided in specialized wards.

## Methods

### Study design

The study was designed as a prospective, observational, single-center study and approved by the Swedish Ethical Review Authority (D-nr 2020-01875). In this ethical approvement, consent was waivered in very severe cases of COVID-19 disease, where patients due to severe medical conditions were not able to give their consent. Patients over 18 years of age, who tested positive for COVID-19 and were considered in need of hospital care, were eligible for inclusion in the study, from May 2020. Here we report on subjects included during the first month.

All patients were included at Capio St Göran’s Hospital, a 300 beds hospital primarily responsible for medical care of patients living in the central area of Stockholm, Sweden. Patients presenting at the Emergency Room with confirmed SARS-CoV-2 infections and in need of hospitalization are admitted either to regular wards with possibility of low-flow oxygen therapy (henceforth “regular wards”) or to wards with possibilities of more advanced ventilation support; either non-invasive ventilation (NIV) in intermediate wards or to the Intensive Care Unit (ICU) where, in addition to NIV, invasive mechanical ventilation support is provided (henceforth “specialized wards”).

Patients suffering from mild to moderate respiratory failure were treated with low-flow oxygen, up to 15 L/min, in regular wards. If this was insufficient, patients were transferred for treatment with high-flow oxygen (HFO) or continuous positive airway pressure (CPAP) in specialized wards. The most severe cases were admitted to the ICU for invasive ventilation support. The attendant physician was responsible for the decision of care level, based on the patient’s need for oxygenation and/or ventilation therapy.

Conventional coagulation tests (CCTs): D-dimer, P-fibrinogen, Activated Partial Thromboplastin Time (APTT), International Normalized Ratio (INR), Antithrombin and Platelet count as well as ROTEM were drawn from all included patients as soon after admission to hospital as possible (median within 1 day after admission). Blood types were added. ROTEM tests were blinded for clinicians, but CCTs were not. Aside from these blood tests, nothing was changed in the routine care of included patients.

Anticoagulant treatment after admission were categorized according to (1) low prophylactic dose Low Molecular Weight Heparin (LMWH) = 75 IE/kg/24 h; (2) high prophylactic dose LMWH = 150 IE/kg/24 h; (3) treatment dose LMWH ≥ 175 IE/kg/24 h; or 4) pre-existing anticoagulant medication, e.g. new oral anticoagulants. The LMWH type administrated was Tinzaparin (Innohep^®^, LEO Pharma, Copenhagen, Denmark).

A ROTEM sigma (Tem Innovations GmbH, Germany) was used for the thromboelastometric analyses. ROTEM sigma is a fully automated system with proven ROTEM technology, where pipetting and test preparation are not required. Analyzing time is 45–60 min for each test. All blood samples were analyzed at St Göran’s Hospital laboratory within 4 h after they were drawn.

**Fig. 1:**
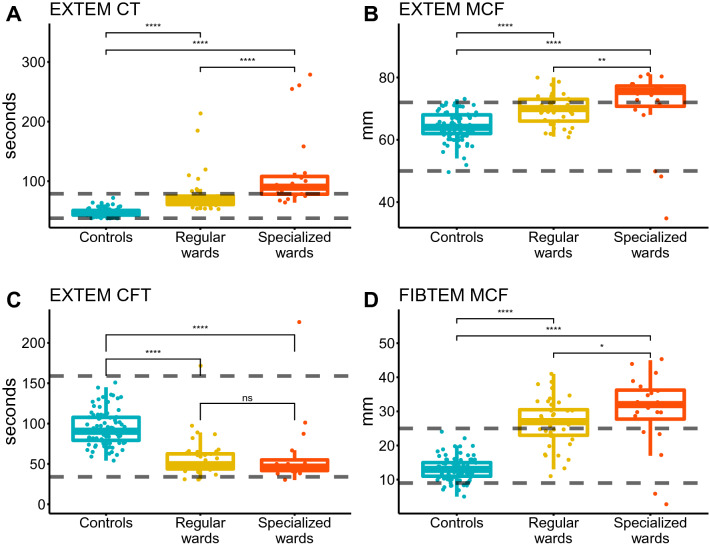
ROTEM data. Dashed horizontal lines are upper and lower reference values. p values were calculated using a two-sided Wilcoxon signed rank test (ns = p > 0.05, ∗p < 0.05, ∗∗p < 0.01, ∗∗∗p < 0.001”, ∗∗∗∗p < 0.0001). In **a** EXTEM Coagulation Time (reference: 38–79 s); **b** EXTEM Maximum Clot Firmness (reference: 50–72 mm); c EXTEM Clot Formation Time (reference: 34–159 s); and **d** FIBTEM Maximum Clot Firmness (reference: 9–25 mm)

**Fig. 2:**
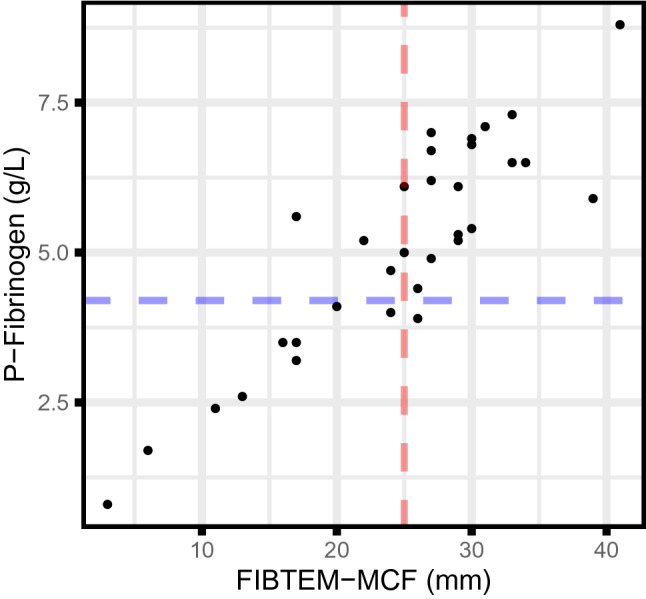
Scatter plots showing ROTEM data and coagulation markers for patients tested during the first day after admission. P-fibrinogen plotted against FIBTEM-MCF. The red vertical line is the upper reference limit for FIBTEM-MCF (25 mm); blue horizontal line upper reference limit for P-Fibrinogen (4.2 g/L)

ROTEM data from healthy blood donors collected before the SARS-CoV-2 pandemic were used as a reference group [14] in the statistical analysis. Reference ranges for ROTEM are added to Figs. 1 and 2.

### ROTEM-tests

Four ROTEM-variables are presented here: (1) extrinsically activated assays with tissue factor (EXTEM); (2) intrinsically activated assays using phospholipid and ellagic acid (INTEM); (3) fibrin-based extrinsically activated assays with tissue factor and platelet inhibitor cytochalasin D (FIBTEM) and (4) intrinsically activated assays with the addition of heparinase (HEPTEM). EXTEM and INTEM provide information about the extrinsic and intrinsic coagulation pathways, respectively. FIBTEM blocks the platelet contribution to clot formation, leaving only the impact of fibrin formation and polymerization. HEPTEM presents coagulation disturbances where the effects of heparin are excluded [15].

Coagulation Time (CT) is the time (in seconds) from test start until an amplitude of 2 mm is reached, giving information about coagulation activation/initiation. Clot Formation Time (CFT) is the time (in seconds) between 2 mm amplitude and 20 mm amplitude, giving information about clot propagation. Maximum Clot Firmness (MCF) is the maximum amplitude (in millimeters) reached during the test, giving information about clot stability. A5 and A10 represent clot firmness (in millimeters) after 5 and 10 min, respectively. LI-30 is the reduction in MCF 30 min after CT (in percent) [15].

A short EXTEM-CFT and an increased EXTEM-MCF and/or FIBTEM-MCF suggest a hypercoagulable state. A prolonged INTEM-CT compared with HEPTEM-CT illustrates a heparin effect.

### Statistical analysis

All continuous variables are presented with medians and interquartile ranges (IQR). Kruskal–Wallis tests were used to test whether ROTEM data for COVID-19 patients differed depending on care level (Regular wards; Specialized wards), or from healthy controls. If the tests were significant, pairwise two-sided Wilcoxon tests were performed. Spearman’s correlation coefficients were calculated to assess associations between variables. p values below 0.05 were considered statistically significant. R version 4.0.0 was used to perform statistical analysis and visualizations. The study was conducted applying the STROBE guidelines.

## Results

60 COVID-19 positive patients were admitted at hospital and included in the analysis. The reference group consisted of 86 healthy controls. Table 1 presents patient characteristics. 40 (67%) of COVID-19 patients were men. Comorbidities were common, where 42% of patients had a prior diagnosis of hypertension and 28% of diabetes. 26 patients (43%) were of non-Caucasian origin.

**Table 1:** Baseline characteristics of COVID-19 patients and healthy controls

	COVID-19			Healthy blood donors
	Regular wards	Specialized wards	Total	
	N = 40	N = 20	N = 60	N = 86
Gender male, N (%)	28 (70%)	12 (60%)	40 (67%)	49 (57%)
Age, years, median (IQR)	61 (51–74)	62 (55–66)	61 (52–71)	49 (37 - 56)
BMI, kg/m^2^, median (IQR)	26 (24–32)	28 (25–32)	27 (24–32)	
Caucasian origin	21 (52.5%)	13 (65%)	34 (57%)	
Non-caucasian origin	19 (47.5%)	7 (35%)	26 (43%)	
Previous thromboembolic disease, N(%)	4 (10%)	2 (10%)	6 (10%)	
Anticoagulant treatment at inclusion, N (%)	8 (20%)	2 (10%)	10 (17%)	
Diabetes, N (%)	8 (20%)	9 (45%)	17 (28%)	
Smoking, N (%)	2 (5%)	3 (15%)	5 (8%)	
COPD/asthma, N (%)	6 (15%)	4 (20%)	10 (17%)	
Hypertension, N (%)	13 (33%)	12 (60%)	25 (42%)	
Cardiovascular disease, N (%)	5 (13%)	4 (20%)	9 (15%)	
Malignancy, N (%)	4 (10%)	4 (20%)	8 (13%)	
Other diseases, N (%)	9 (23%)	6 (30%)	15 (25%)	
Days with COVID symptoms at inclusion, median (IQR)	10 (7–14)	12 (9–16)	11 (7–14)	
Days at hospital at inclusion, median (IQR)	1 (1–2)	2.5 (1–5)	1 (1–2)	
Thrombosis at inclusion, N (%)	2 (5%)	4 (20%)	6 (10%)	
Anticoagulant prophylaxis before ROTEM analysis, N (%)	21 (53%)	15 (75%)	36 (60%)	

Blood groups, N = 58	N (%)
A	19 (33%)
B	6 (10%)
AB	3 (5%)
O	30 (52%)

	Regular wards	Specialized wards	
Non-O	21 (75%)	7 (25%)	28 (48%)
O	18 (60%)	12 (40%)	30 (52%)

*IQR* interquartile range, *BMI* body mass index, *COPD* chronic obstructive pulmonary disease

10 patients (17%) had ongoing anticoagulant treatment at inclusion (6 patients on Direct oral anticoagulants (DOAC), 1 patient on Warfarin, 3 patients on LMWH). 49 patients (82%) received anticoagulant treatment after admission (29 patients had low dose LMWH, 9 patients had high dose LMWH, 6 patients had treatment dose LMWH and 5 patients continued their regular DOAC treatment, none had Warfarin). No standard monitoring of anticoagulant treatment guided by therapeutic goals was performed. Among the 49 patients receiving anticoagulant treatment after admission, this was administrated prior to ROTEM-analysis in 36 patients (60%), whereof 19 received low prophylactic dose LMWH, 7 high prophylactic dose LMWH, 5 treatment dose LMWH and 5 patients received DOAC.

In 58/60 patients main blood types were determined. The blood type distribution is presented in Table 1: A (33%), B (10%), AB (5%) and O (52%). A larger proportion of our patients had blood type O compared with in the Swedish population (38%). Among the O blood group patients, 40% needed ventilation support in specialized wards compared with 25% of patients of non-O blood groups.

Table 2 depicts laboratory test results in COVID-19 patients and healthy controls. *D*-*dimer* was increased in patients in specialized wards compared with patients in regular wards. *P*-*fibrinogen* was increased in patients in both regular and specialized wards compared with controls, but there were no significant differences observed between care levels. The data are also visualized in *Additional file 1, Figures AF1* and *AF2*.

**Table 2:** Laboratory test results for COVID-19 patients and healthy controls

	COVID-19			Healthy controls	Healthy controls versus COVID-19 patients (p)	Regular wards versus Specialized wards (p)
	Regular wards	Specialized wards	Total			
	N = 40	N = 20	N = 60	N = 86		
Covid-positive, verified	40 (100%)	20 (100%)	60 (100%)			
D-dimer, mg/L, median (IQR)	0.6 (0.5–1.0)	1.5 (0.7–4.0)	0.7 (0.5–1.5)			0.002
Hemoglobin, g/L, median (IQR)	137 (125–148)	123 (110–128)	129 (120–141)	141 (134–150)	<0.001	<0.001
Platelet count, 10^9^/L, median (IQR)	212 (175–259)	252 (206–341)	221 (181–287)	257 (229–283)	0.005	0.094
APTT, sec, median (IQR)	26 (24–27)	26 (25–30)	26 (24–29)	33 (31–36)	<0.001	0.891
INR, median (IQR)	1.0 (1.0–1.1)	1.0 (1.0–1.1)	1 (1–1.1)	1.0 (1.0–1.1)	0.107	0.843
P-fibrinogen, g/L, median (IQR)	5.4 (4.3–6.5)	6.8 (4.8–7.6)	5.7 (4.3–6.9)	2.7 (2.4–3.0)	<0.001	0.125
Antithrombin, kIE/L, median (IQR)	1.0 (0.9–1.1)	1.0 (0.9–1.1)	1.0 (0.9–1.1)			0.836
EXTEM-CT, sec, median (IQR)	70 (61–75)	90 (78–108)	74 (64–90)	47 (43–51)	<0.001	<0.001
EXTEM-CFT, sec, median (IQR)	49 (44–63)	46 (42–55)	49 (43–63)	91 (79–101)	<0.001	0.485
EXTEM-A5, mm, median (IQR)	51 (47–54)	57 (54–60)	53 (48–58)			0.009
EXTEM-A10, mm, median (IQR)	61 (57–65)	68 (63–70)	63 (59–68)			0.006
EXTEM-MCF, mm, median (IQR)	70 (66–73)	76 (71–77)	71 (68–75)	64 (62–68)	<0.001	0.009
EXTEM-LI30, %, median (IQR)	100 (100–100)	100 (100–100)	100 (100–100)			0.632
INTEM-CT, sec, median (IQR)	190 (173–202)	183 (175–195)	187 (173–202)	163 (157–170)	<0.001	0.588
INTEM-CFT, sec, median (IQR)	60 (50–73)	46 (40–-59)	56 (45–71)	63 (57–72)	0.001	0.041
INTEM-A5, mm, median (IQR)	47 (42–51)	54 (49–59)	49 (45–54)			0.014
INTEM-A10, mm, median (IQR)	58 (54–61)	64 (60–68)	60 (55–64)			0.010
INTEM-MCF, mm, median (IQR)	67 (63–69)	72 (68–75)	67 (64–72)	66 (64–69)	0.050	0.006
INTEM-LI30, %, median (IQR)	100 (100–100)	100 (100–100)	100 (100–100)			1.000
FIBTEM-MCF, mm, median (IQR)	27 (23–31)	32 (28–36)	29 (24–33)	13 (11–15)	<0.001	0.036
HEPTEM-CT, sec, median (IQR)	188 (180–203)	179 (173–191)	182 (175–199)			0.089

*IQR* interquartile range, *APTT* activated partial thromboplastin time, *INR* international normalized ratio

For EXTEM-CT, the Kruskal–Wallis test showed a significant difference between the groups (H(2) = 97.1, p < 0.001). Post-hoc pairwise Wilcoxon tests indicated that COVID-19 patients (both care levels) had significantly longer CT compared with healthy controls (p < 0.001) and that subjects treated at specialized wards had longer CT compared with subjects treated at regular wards (p < 0.001) (Fig. 1a, Table 2).

For EXTEM-MCF, the Kruskal–Wallis test displayed a significant difference between the groups (H(2) = 39.3, p < 0.001). Post-hoc pairwise Wilcoxon tests showed that COVID-19 patients (both care levels) had significantly increased MCF compared with healthy controls (p < 0.001) and that subjects treated at specialized wards had increased MCF compared with subjects treated at regular wards (p < 0.01) (Fig. 1b, Table 2). This was true also for A5 and A10 (Table 2).

For EXTEM-CFT, the Kruskal–Wallis test revealed a significant difference between the groups (H(2) = 64.8, p < 0.001). Post-hoc pairwise Wilcoxon tests showed that COVID-19 patients (both care levels) had significantly shorter CFT compared with healthy controls (p < 0.001) (Fig. 1c, Table 2).

For FIBTEM-MCF, the Kruskal–Wallis test showed a significant difference between the groups (H(2) = 79.5, p < 0.001). Post-hoc pairwise Wilcoxon tests indicated that COVID-19 patients (both care levels) had significantly increased MCF compared with healthy controls (p < 0.001) and that subjects treated at specialized wards had increased MCF compared with subjects treated at regular wards (p = 0.04) (Fig. 1d, Table 2).

EXTEM-/INTEM-LI30 were both median 100 (IQR 100-100), which illustrate an absence of enhanced fibrinolytic activity in the COVID-19 patients.

In order to evaluate the impact of heparin on our test results, the differences of INTEM-CT and HEPTEM-CT were assessed in patients receiving LMWH prior to ROTEM-analysis (N = 31). Among the 31 patients to whom LMWH was administrated prior to ROTEM analysis, 19 had INTEM-CT longer than HEPTEM-CT, 10 had INTEM-CT shorter than HEPTEM-CT and in 2 patients there were no differences observed. When inspected in a Bland–Altman plot, no obvious pattern was observed in patients on low- or high-dose LMWH (N = 26; INTEM-CT median 187 (IQR 177–206) seconds; HEPTEM-CT 183 (179–203) seconds and difference score of 1 (− 4 to 7), paired Wilcoxon test, p = 0.28). However, when patients receiving treatment dose LMWH (N = 5) were added, the INTEM-HEPTEM differences became significant (INTEM-CT 187 (179–207) seconds; HEPTEM-CT 182 (179–201) seconds and difference score of 2 (− 3 to 8), paired Wilcoxon test, p = 0.044) (*Figure AF3*).

We observed a significant correlation between P-Fibrinogen and FIBTEM-MCF (r_s_ = 0.84, p < 0.001) (Fig. 2).

## Discussion

Our results show that patients with COVID-19 related mild to severe acute respiratory failure treated at St Göran’s Hospital in May 2020, had laboratory results indicating coagulopathy early after admission, with significant differences in ROTEM variables between wards with normal and increased need of ventilation support as well as compared with healthy controls; longer EXTEM-CT, shorter EXTEM-CFT and increased EXTEM-MCF and FIBTEM-MCF, i.e. prolonged activation of coagulation, shortened clot propagation and, notably, a pronounced clot firmness. This means that the patient’s clots take longer to begin to form, but once commenced, the clots rapidly increase their strength and ultimately have greater strength than healthy subject’s clots have. Taken together, these findings suggest a hypercoagulative state in hospitalized COVID-19 patients, which could possibly be associated with a higher risk of a poor prognosis.

We cannot exclude that part of the prolonged EXTEM-CT that we observe in COVID-19 patients, indicating a prolonged initiation of clotting, could be driven by a heparin effect as a majority of patients had received anticoagulant treatment before ROTEM analysis.

The reduced EXTEM-CFT and increased EXTEM-/FIBTEM-MCF, illustrating an accelerated increase in clot strength, are in line with elevated P-fibrinogen levels and increased platelet activation observed in severe COVID-19 patients [16], related to the massive inflammatory response associated with the disease.

In this prospective, observational study, the aim was to describe the characteristics of patients admitted to hospital due to COVID-19 and to assess the level of coagulopathy using ROTEM analysis. Previous studies have shown deranged coagulation laboratory values in critically ill patients [12, 13]. However, the level of coagulopathy in less severely ill patients early after admission to hospital is not well known.

During the months since COVID-19 was classified as a pandemic, evidence has emerged indicating coagulopathy and thrombosis as crucial explanations to why some patients develop severe illness [3, 5]. The pattern of higher EXTEM-MCF and FIBTEM-MCF in more severely respiratory ill patients in our data strengthens the hypothesis that the atypical pulmonary symptoms observed in COVID-19 patients may partly be caused by thromboembolism impairing lung perfusion.

Increased D-dimer and P-fibrinogen levels have been reported to predict poor clinical outcome in COVID-19 patients [9, 10]. However, both these tests have caveats: the D-dimer blood test is nonspecific and may be increased in a variety of conditions including malignancy, inflammation and infection [17]; fibrinogen is an acute phase reactant, and a high level of P-fibrinogen may reflect a patient with an increased inflammatory profile, which itself may amplify the effects of other cardiovascular risk factors [18]. Furthermore, conventional coagulation tests (CCTs) are limited by their inability to assess clot strength, fibrinogen functionality and fibrinolysis [19]. Conversely, ROTEM provides a more rapid and comprehensive assessment of the whole blood clot formation allowing for a complete view of the entire coagulation cascade, and has been shown a better tool for monitoring coagulation profiles than CCTs [20, 21].

A majority of our included patients (67%) were men. That is in line with previous studies, showing that men seem to be more prone than women to get infected with SARS-CoV-2, which may be due to gender-related differences in innate immunity and hormone levels [22, 23]. From a coagulation perspective, this is interesting, as several studies have indicated differences in coagulation factor levels between men and women [24], which may possibly have an impact on the risk of thromboembolic events.

Earlier findings have suggested that non-O blood types are associated with a higher risk of acquiring COVID-19 than blood type O [25]. On the contrary, we observed a high proportion of patients with blood group O (52%) compared with the lower prevalence in the Swedish population (38%) (personal communication T Dahlén, 2020, based on the SCANDAT database). A possible explanation may be that 43% of our patients are of non-Caucasian origin (mostly from the Middle East and Africa) where blood type O is more frequent.

As depicted in Fig. 2, FIBTEM-MCF and P-fibrinogen were highly correlated in COVID-19 patients, whereas EXTEM-MCF data did not seem to correlate with D-dimer values at an early disease stage. This discrepancy is interesting and may have consequences. If individuals at risk of developing COVID-19 related thrombosis are identified at an early stage, enhanced prophylaxis with LMWH may decrease mortality in this group of patients [9, 26]. It is feasible that ROTEM may be applied for this purpose. Whether the ROTEM data will have better predictive characteristics compared with CCTs do remain to be shown.

### Limitations

A large proportion of patients had received anticoagulant treatment before ROTEM analysis, usually low dose LMWH, which may have to some degree counteracted hypercoagulation and may have influenced our results. However, out of the variables we consider most clinically relevant (reported in Fig. 1) only for EXTEM-CT could the reported differences be expected to be partly explained by a heparin effect.

Some patients admitted for COVID-19 during this period were not tested using ROTEM, and a few patients that were included were tested later than the first day after admission. The main reasons for this were: (i) Due to clinical duties the physicians responsible for the study were not always able to scan the wards for new patients on a daily basis (ii) more importantly, limitation of the testing equipment, with the possibility to run a maximum of 6 tests every 4 h. Still, we believe that the included patients are a representative cross-section of the COVID-19 patients treated at St Göran’s Hospital during the study period.

Lastly, the healthy controls used as a reference group in the statistical analysis were younger and had more females, which may have had an impact on the results.

## Conclusions

Our results indicate a hypercoagulable state already on admission in COVID-19 positive patients in need of hospitalization, as shown by elevated values of EXTEM-MCF and FIBTEM-MCF. This pattern was more pronounced in patients with more severe symptoms. Coagulopathy being present early in the disease course suggests ROTEM as a potential predictor of thromboembolic complications and mortality in COVID-19 disease.

## Electronic supplementary material

Below is the link to the electronic supplementary material.

Supplementary material 1 (DOCX 47884 kb)

## Data Availability

The data that support the findings of this study are openly available in *Swedish national dataservice* at: 10.5878/wh80-0w17.

## Abbreviations

COVID-19: Corona virus disease 2019
SARS-CoV-2: Severe acute respiratory syndrome coronavirus 2
ICU: Intensive care unit
MCF: Maximum clot firmness
ROTEM: Rotational thromboelastometry
NIV: Non-invasive ventilation
HFO: High-flow oxygen
CPAP: Continuous positive airway pressure
CCT: Conventional coagulation test
APTT: Activated partial thromboplastin time
INR: International normalized ratio
LMWH: Low molecular weight heparin
EXTEM: Extrinsically activated thromboelastometry
INTEM: Intrinsically activated thromboelastometry
FIBTEM: Fibrin-based extrinsically activated thromboelastometry
HEPTEM: Intrinsically activated thromboelastometry with heparinase
CT: Coagulation time
CFT: Clot formation time
MCF: Maximum clot firmness
A5: Clot amplitude at 5 min
A10: Clot amplitude at 10 min
LI-30: Lysis Index at 30 min
IQR: Interquartile range
DOAC: Direct oral anticoagulants
