# Supplementary material for: Rotational thromboelastometry results are associated with care level in COVID-19
Source: J Thromb Thrombolysis. 2020 Oct 17;51(2):437–45. doi: 10.1007/s11239-020-02312-3 (PMC7568025; doi:10.1007/s11239-020-02312-3)
Supplement: Supplementary file 1 — Supplementary material 1 (DOCX 47884 kb) [file 11239_2020_2312_MOESM1_ESM.docx]

**Rotational Thromboelastometry results are associated with care level in COVID-19.**

*Additional file*


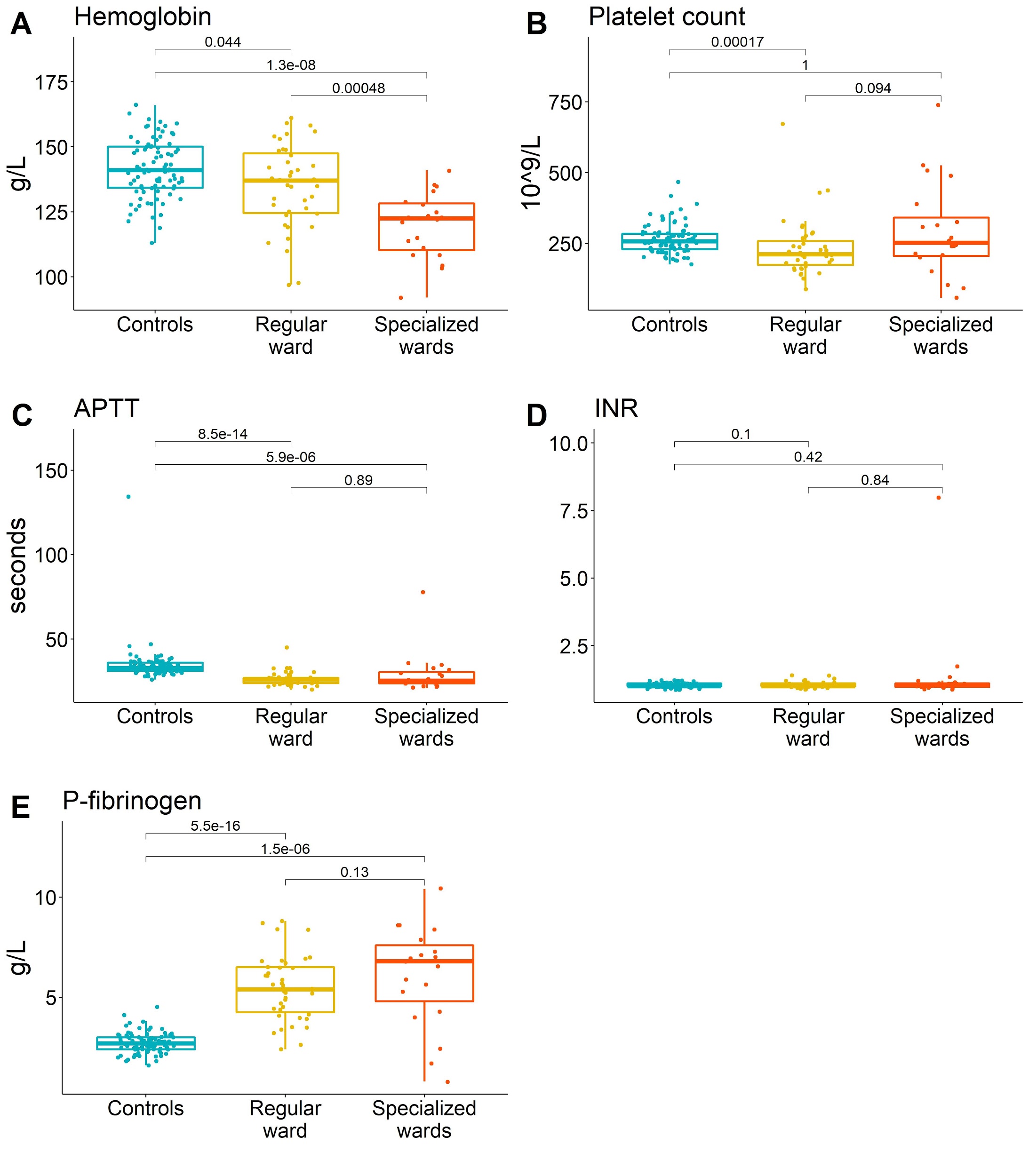


**Figure AF1.** Visualization of data for blood tests presented in table 2. P-values calculated using two-sided Wilcoxon signed rank test. In A) Hemoglobin; B) Platelet count; C) Activated Partial Thromboplastin Time D) International Normalized Ratio E) P-fibrinogen


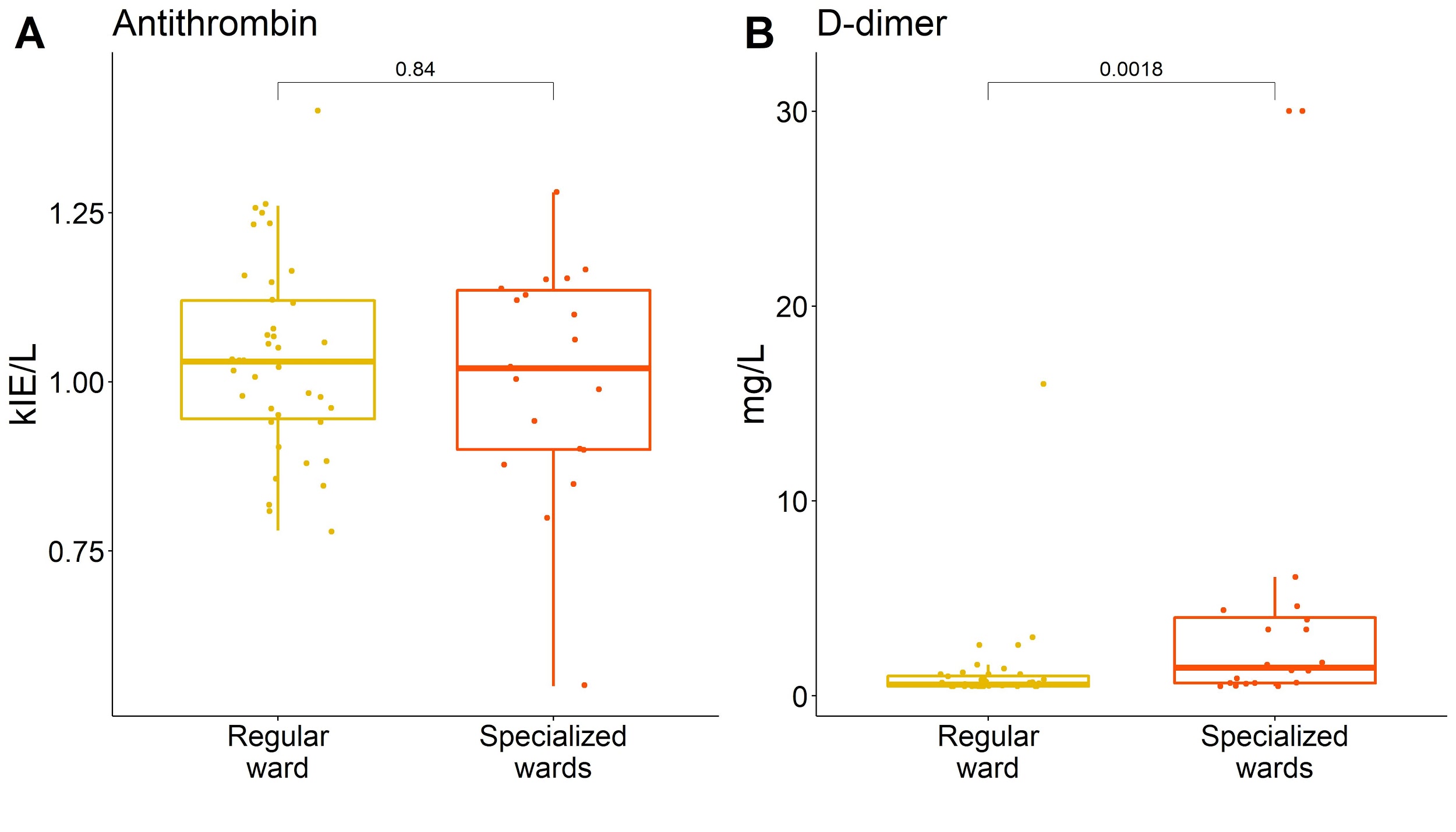


**Figure AF2.** Visualization of data for blood tests presented in *Table 2*. For tests presented in this figure no data from healthy controls were available. P-values calculated using two-sided Wilcoxon signed rank test. In A) Antithrombin; B) D-dimer

**Figure AF3.** Bland-Altman plot to evaluate the agreement between INTEM-CT and HEPTEM-CT among patients receiving LMWH prior to ROTEM analysis. In the figure one outlier that received high dose LMWH was removed (difference score of 78 and average CT score of 360). Data from this subject was used in statistical calculations.
